# Effects of Process Parameters in Thermoforming of Unidirectional Fibre-Reinforced Thermoplastics

**DOI:** 10.3390/polym16020221

**Published:** 2024-01-12

**Authors:** Johannes Winhard, Daisy Nestler, Lothar Kroll

**Affiliations:** Department of Ligthweight Structures and Polymer Technology, Faculty of Mechanical Engineering, Chemnitz University of Technology, 09111 Chemnitz, Germany

**Keywords:** thermoplastic matrix composite, fibre-reinforced polymer, thermoforming, design of experiments, basalt fibres, BF/PA6

## Abstract

Process-induced defects during thermoforming are widespread problems in laminate manufacturing. The aim of this study is to describe the effects of holding time and pressure on several properties of the manufactured laminate. A design of experiments is performed, followed by an analysis of variance to examine significant effects. Subsequently, a regression model is created to predict the laminate’s properties, which is also validated. A significant interaction between holding time and pressure is determined for the resulting tensile strength and elongation at break with a *p*-value of $1.52\;\cdot \;10^{-16}$ and 0.02, respectively. The highest values of tensile strength and elongation at break are found for low settings of holding time and pressure. The fibre volume fraction is not affected by the process parameters. As holding time and pressure increase, significant fibre misalignment takes place, leading to a decrease of the mechanical properties. The regression model corresponds well with the validation and a tensile strength of 1049 MPa with an elongation at break of 2.3% is reached.

## 1. Introduction

Fibre-reinforced thermoplastics (FRTP) are primarily used in the transportation sector, such as automobiles and aerospace, relying on lightweight structures combined with high strength and stiffness [1]. The manufacturing takes place at temperatures near the melting point of the used thermoplastic matrix to reduce its viscosity and enable sufficient impregnation of the fibres. Common melt viscosity values for thermoplastics are 10^2^–10^4^ Pa·s, which are much higher compared to those of epoxy polymers during impregnation (10^−1^–10^1^ Pa·s) [2,3]. As a result, different manufacturing processes need to be considered, and a fundamental understanding of the respective process parameters is crucial. Furthermore, a thermoplastic matrix offers the possibility of short cycle times due to the use of semi-finished materials, such as prepregs (pre-impregnated material), organo sheets, or unidirectional fibre-reinforced tapes (UD-tapes) [4,5]. This paper focuses on UD-tapes, which can be oriented in any load direction, allowing for targeted fulfilment of current load requirements.

The thermoforming process for UD-tapes and hybrid laminates is widely used and has been described in detail in many sources. In general, thermoforming involves a stamping process executed under a specific pressure, time and temperature just below the melting point of the matrix material. The UD-tapes are stacked into the negative die, with each layer having a specific fibre orientation. Then the tool closes by moving down the positive die and the thermoforming process executes with defined process parameters [4,6,7,8]. Consequently, understanding the impact of process parameters on the quality and mechanical properties of the manufactured laminate is of significant interest. Nonetheless, thermally induced residual stress and process-induced defects, such as fibre misalignment, wrinkling and folding, are common challenges when thermoforming thermoplastic prepregs [6,9,10,11]. These defects can lead to a decrease in mechanical properties, unintended plastic deformation, or premature material failure. The compressive strength of fibre-reinforced plastics (FRP) in particular is drastically affected even by small fibre misalignments. This is assumed for the first time by Wisnom [12] with a simplified model for carbon fibres and epoxy resin showing a decrease to 26% of the original compressive strength for a fibre misalignment angle of $3^{\circ }$. Furthermore, the tensile strength decreases sharply with an increasing off-axis angle [13,14] as well. This is caused by the fibre-parallel shear stress, whose critical value for failure of the laminate is much lower than shear strength perpendicular to the fibres or tensile strength in any direction [15,16] and illustrates the sensitivity of the mechanical properties to the fibre orientation. Numerous strategies for modelling various effects on the impregnation process have been developed [17,18,19,20]. However, each simulation is constrained by simplifications, limitations, or a focus on specific aspects due to the complex and interdisciplinary nature of the problem [21]. Manson et al. [22] emphasized the significance of uniform pressure distribution for consistent laminate impregnation. Their study found that eight-ply UD-tapes subjected to uniform pressure across the entire laminate exhibited negligible void content, regardless of cooling rates or annealing conditions. Furthermore, annealing increased the crystallinity of the polyether ether ketone (PEEK) matrix [22], resulting in reduced composite fracture toughness [23,24]. Christmann et al. [18] developed a thermoforming model for FRTP based on the so-called B-Factor model. This model demonstrated identical impregnation quality for different parameter settings. Additionally, model validation indicated that varying pressure settings does not affect impregnation significantly for rapid processing times. Lower pressure settings appeared advantageous for impregnation quality and yielded higher interlaminar shear strength values as well [25]. Conversely, various observations [10,26] suggest that higher pressure leads to improved part quality, particularly concerning the surface roughness.

In the present work, a design of experiments (DOE) is conducted to investigate the effects of pressure and holding time on the tensile strength, elongation at break, and compaction behaviour of UD-tapes with basalt fibres (BF) within a polyamide 6 (PA 6) matrix. Furthermore, macroscopic misalignment of the fibres resulting from the thermoforming process is discussed, and a validation of the developed model is performed.

## 2. Materials and Methods

### 2.1. Materials and Samples

The material system examined in this study consists of UD-tape comprising basalt fibres and a PA 6 matrix from Cetex Institut gGmbH (Chemnitz, Germany), with a thickness of 0.16 mm. The fibre volume fraction (FVF) of 62% is determined following DIN EN ISO 1172 [27]. Square laminates measuring 260 mm × 260 mm are produced using the Collin P 300 P/M (COLLIN Lab & Pilot Solutions GmbH, Maitenbeth, Germany) hot plate press, in accordance with DIN 65672 [28] standards, resulting in a six-ply laminate oriented of [0]_6_. Each specimen is cut out using a waterjet and then subjected to accelerated conditioning (DIN EN ISO 1110 [29]). In this standard, the specimen is stored in a climate chamber at (70 ± 1) °C to accelerate the moisture absorption of PA 6. The mass of the specimen is measured frequently after specific periods of time and is repeated until the measurements of three consecutive cycles differ by less than 0.1% [29]. The tensile tests are performed according to DIN EN ISO 527-5 [30].

**Figure 1:**
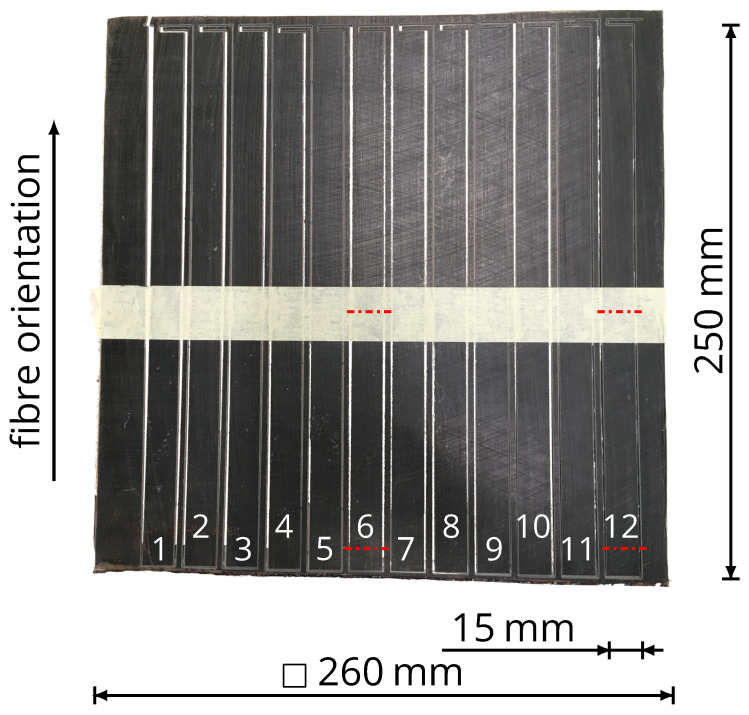
Square laminate with cut-out specimens 1–12, each with dimensions of 15 mm × 250 mm, for tensile tests. The dash-dotted lines indicate the positions of the performed light microscopy and the adhesive tape across the laminate prevents the individual specimens from ripping out unintentionally.

A single laminate allows for the creation of twelve specimens (Figure 1), out of which eight are designated for the tensile tests, two for FVF measurement, and two for quality assessment of the laminate. Additionally, two specimens serve as backups in case of damage during waterjet cutting. Multiple light microscopic images are captured from the centre, corner and edges of each laminate, perpendicular to the fibres, to evaluate impregnation and compaction quality.

### 2.2. Manufacturing Process

**Figure 2:**
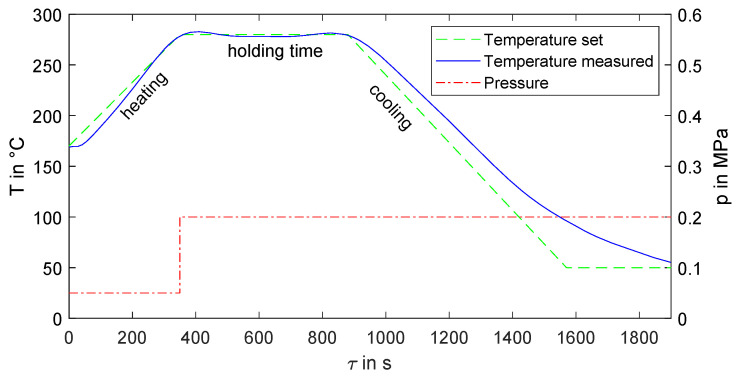
Program example of a thermoforming process with the set parameters of temperature *T*, pressure *p* and measured temperature over the process time τ during thermoforming with a holding time of 530 s.

An illustrative instance manufacturing processes is depicted in Figure 2. The stacked UD-tapes undergo preheating to 170 °C and are then heated up to 280 °C at a rate of 20 K/min. Upon attaining the designated processing temperature, the pressure increases to the predetermined value, maintaining a constant temperature for the specified holding duration. Subsequently, the laminate is gradually cooled at a rate of 20 K/min until reaching 50 °C. The pressure remains constant until the conclusion of the process. The observed temperature closely aligns with the predetermined values. A marginal temperature disparity is observable at the onset of the heating phase. During the entire cooling process, the temperature differences are somewhat more pronounced, although the cooling rate is effectively maintained.

### 2.3. Statistical Methods

A full factorial design of experiments is executed, encompassing various holding times ranging from 60 s to 1000 s. These durations are maintained at a consistent temperature of 280 °C, alongside varying pressures from 0.2 MPa to 2.0 MPa. Each parameter is set at three distinct levels, which results in a total of nine individual laminates. The configuration and assignment of process parameters to each laminate are detailed in Table 1.

The primary objectives of this DOE encompasses the evaluation of tensile strength, elongation at break and FVF. The hypothesis is that as the holding time and pressure increase, there will be a tendency for fibre misalignment. Consequently, the fibres may deviate from being parallel to the applied load direction, leading to a reduction in tensile strength. Moreover, a greater degree of fibre misalignment towards the dominant load direction is anticipated to cause a more pronounced decline in elongation at break. This is attributed to potential obstacles in transverse contraction and issues stemming from low adhesion.

After mechanical testing of the samples, an analysis of variance (ANOVA) is conducted to identify significant effects. If detected, main effects and interactions of the manipulated factors are elucidated. Subsequently, a regression model is formulated and its accuracy is validated. Both the ANOVA and the regression model employ statistical tools from MATLAB (version 9.12) [31].

## 3. Results and Discussion

### 3.1. Macroscopic Fibre Misalignment and Compaction

**Figure 3:**
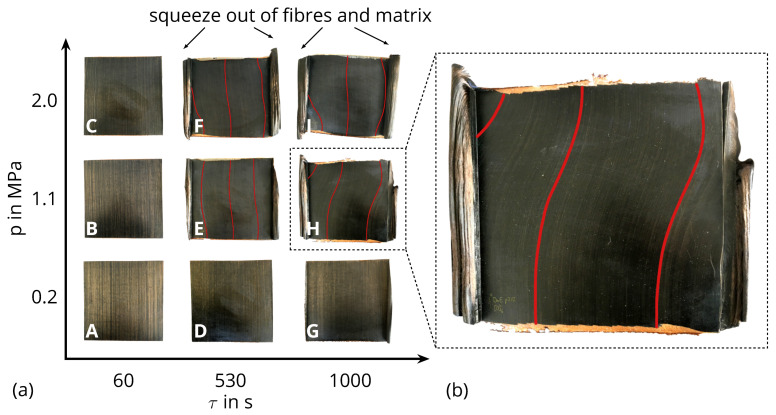
(**a**) Laminates (**A**)–(**I**) manufactured via thermoforming grouped according to the process parameters described in Table 1. The lines indicate the misalignments of the fibres within single laminates. (**b**) Close-up of laminate H.

Figure 3 depicts the produced laminates, categorized based on the process parameters outlined in Table 1 as well as a close-up of laminate H for a better visibility of the fibre misalignment of several laminates. When the holding time or pressure increases, while the other parameter remains at a low level, a minimal amount of fibre and matrix extrusion occurs (laminate G), or no extrusion transpires at all (laminates A–D). In contrast, when both parameters are elevated simultaneously, a substantial extrusion and fibre misalignment are observed across the entirety of laminates E, F, H, and I. Consequently, this leads to the formation of a curled structure composed of fibres and matrix along the edges of the laminates, parallel to the initial 0°-axis of the fibres. Furthermore, areas devoid of fibres emerge at the edges perpendicular to the orientation of the fibres (Figure 3). These outcomes underscore the presence of an interaction between the process parameters, namely the holding time and pressure.

**Figure 4:**
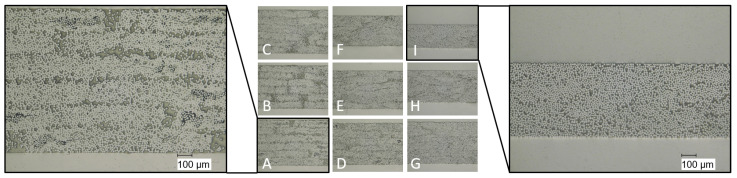
Optical micrographs of the laminates with detailed views of laminate A and I.

The optical micrograph captured from the centre of all laminates (Figure 4) reveals minor discrepancies across the samples A–C, D and G. In the case of laminates A–C and D, distinct layers from the original UD-tapes, or even the primary fibre rovings within each layer, are clearly discernible. Occasional microscopic voids (depicted as black regions in Figure 4) are only observable within the initial fibre rovings of laminates A and D. Consequently, the impregnation quality for all laminates is excellent. The compaction process for laminates E, F, G, H, and I has progressed to the extent that fibres from individual layers have merged into adjacent ones, and thus no separation of individual layers is recognisable. The laminates A–D and G exhibit comparable thicknesses, ranging from 0.90 mm to 0.97 mm. In contrast, laminates E, F, H, and I display noticeable thickness variations, with laminate I measuring only 52% of the thickness of laminate A. This divergence is a direct consequence of the pronounced material extrusion detailed earlier and evident in Figure 3. Once more, significant differences are recognisable when both process parameters are concurrently altered, as opposed to modifying only a single parameter. This serves to reinforce the assumption of an interaction between the process parameters.

### 3.2. Fracture Types and Mechanical Properties

The tensile tests are conducted following DIN EN ISO 527-5 [30]. It is noteworthy to mention that the thickness of laminates F, H, and I falls below the prescribed range stipulated by the standard, as evident from Figure 4. These mentioned laminates do not align with the standardized method’s specifications. However, it is worth noting that despite this variance, the standard deviation of the tensile strength remains consistent with the values observed in samples adhering to the standard.

**Figure 5:**
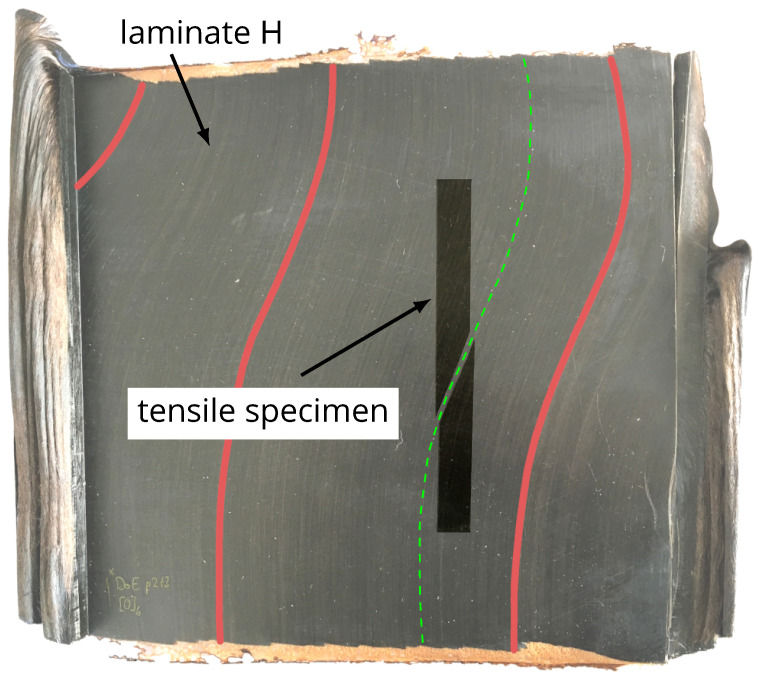
Transparent foreground: image of laminate H with indicators of fibre misalignment (full lines) before tensile tests; background: image of tensile specimen of laminate H after tensile tests at its original position with the local fibre misalignment of laminate H (dashed line).

The laminates A–C, D, and G show typical fracture behaviour of UD-laminates with breaking perpendicular to the fibre orientation and splicing of the specimens at an applied tension in the $0^{\circ }$ direction. In contrast, the tensile samples of laminates E, F, H, and I, with their noticeable fibre misalignment (Figure 3), fail along the path of the fibres with the biggest angular difference to the initial $0^{\circ }$ orientation of the lay-up. Figure 5 shows the image of laminate H and an exemplary tensile specimen in their original position on the plate after the tensile test. The dashed line indicates the local fibre misalignment, which matches well with the fracture of the specimen. All samples of laminates E, F, H, and I follow this fracture behaviour.

**Figure 6:**
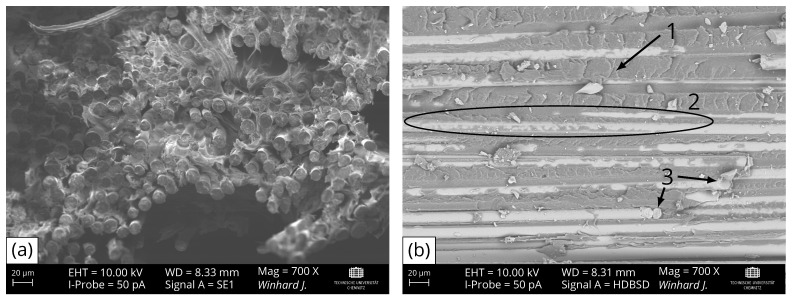
(**a**) Fracture surface of laminate A imaged via secondary electron detector: mainly fibre breakage and fibre pull-out visible; (**b**) fracture surface of laminate H imaged via back-scatter detector: fibre-parallel fracture with predominate (1) matrix fracture and (2) interfacial debonding with matrix residues on fibre surfaces as well as a small amount of (3) fibre breakage visible.

Selective electron microscopy (SEM) images of the resulting fracture surfaces show different fracture types for laminates with macroscopically different failure behaviour (Figure 6). The macroscopic splicing of the tensile specimens in laminate A is accompanied by clear fibre breakage and fibre pull-out (Figure 6a). For imaging the fracture surface of laminate H (Figure 6b), the back-scatter detector is used to distinguish more clearly between matrix material and fibres. Due to the higher atomic numbers of metallic elements (e.g., silicon, aluminium, iron and calcium) of basalt fibres, they appear brighter than to the non-metallic elements (e.g., carbon and nitrogen) of PA6. The present fracture type is inter-fibre failure due to matrix fracture and interfacial debonding with matrix residues on the fibre surfaces, which underlines a good fibre–matrix adhesion. Only a small amount of fibre breakage is visible. It can also be seen that the resulting fracture surface follows the local fibre orientation. This failure mode occurs due to the transformation of the initially normal load on the specimen into a localized stress state, resulting in shear-induced failure. It has been proven theoretically and experimentally that for unidirectional laminates, the local shear load along the fibre direction is the most critical criterion in this case and leads to failure of the laminate [15,16,32]. Additionally, the fracture structure of the matrix at position 1 in Figure 6b indicates a failure caused by shear load as well due to initially formed microcracks caused by normal load, which subsequently unite to several inter-fibre cracks, designated as “hackles” [33,34]. Therefore, the laminates A–C, D and G with a splicing fracture behaviour fail due to normal stress, whereas the laminates E, F, H, and I follow a failure caused by shear stress to a fibre-parallel fracture.

**Figure 7:**
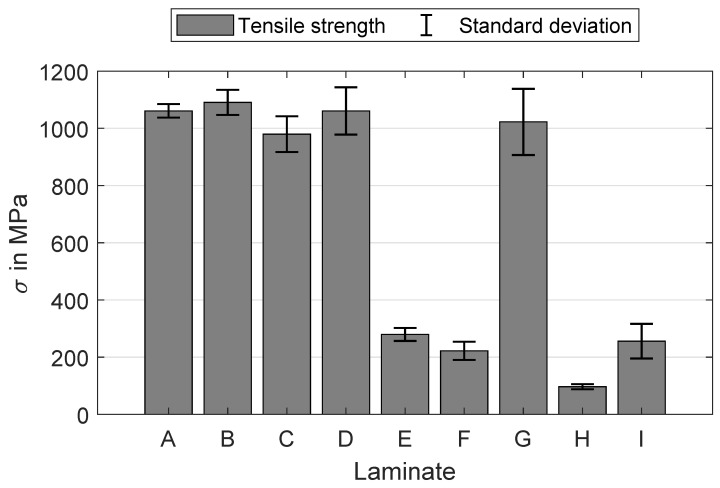
Tensile strength σ in MPa and the corresponding standard deviation for each manufactured laminate A–I.

**Figure 8:**
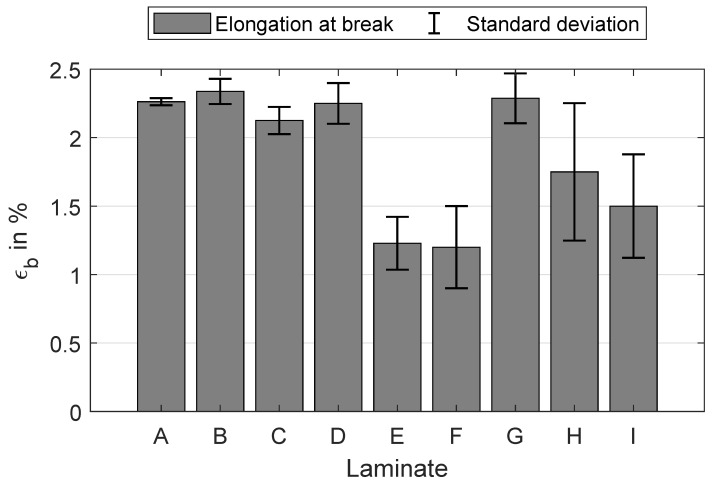
Elongation at break $\epsilon_{b}$ in % and the corresponding standard deviation for each manufactured laminate A–I.

The results for tensile strength are shown in Figure 7. The highest values are attained by laminates A, B and D with approximately 1050 MPa. A slight decrease in mean tensile strength is observed for laminates C and G, with about 1000 MPa. For these samples, only one parameter is varied while keeping the other at its lowest setting. However, when both process parameters are concurrently adjusted, the tensile strength decreases suddenly below 300 MPa for laminates E, F, H, and I. This trend aligns with the laminates where fibre misalignments are clearly visible (Figure 4). The tensile stress applied to the cross-section of the specimen leads to shear stresses at the points of fibre misalignment whose critical value for fracture is well below the tensile strength in the fibre direction. Therefore, the measured tensile strength of laminates E, F, H, and I is much less compared to the other laminates without fibre misalignment, despite the good impregnation of all laminates (Figure 4). A similar trend is noticeable in the results for elongation at break (Figure 8), but to a lesser extent in a comparison of tensile strength. Furthermore, the variance of the elongation at break seems to rise when the settings are incrementally increased. This variance ranges from (2.26±0.03)% for laminate A up to a 16 times higher standard deviation for laminate H with (1.75±0.50)%. A reason for the higher variances of the laminates E, F, H, and I is probably the slightly different maximum deviations of the fibre orientation from the 0°-axis within a laminate. It is known that the elongation at break is significantly lower in pure transverse tensile than in transverse shear and longitudinal tensile [15,35].

The results from the tensile tests substantiate the assumption of diminishing tensile strength and elongation at break, attributed to fibre misalignment in laminates E, F, H, and I. It strongly suggests the existence of a notable interaction among the process parameters. After checking for normal distribution of the target values, an ANOVA is carried out to determine the possible detectability of these effects.

### 3.3. Normal Distribution of Sample Values

**Figure 9:**
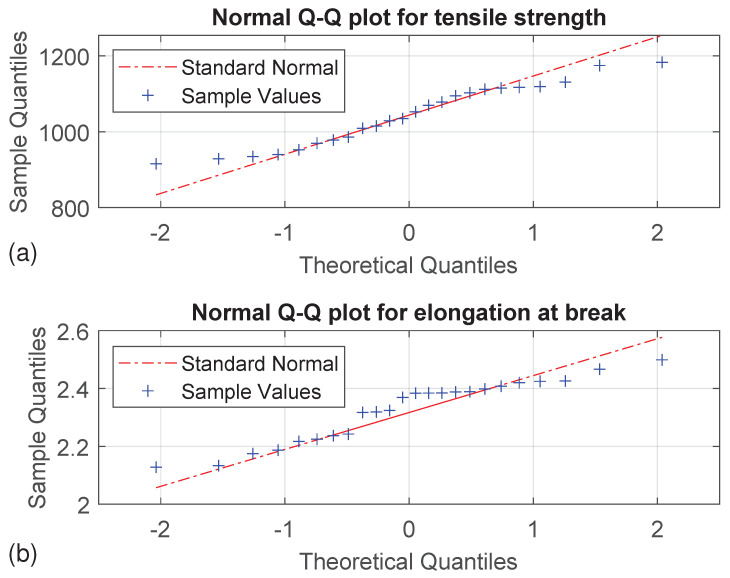
Q-Q plots of (**a**) tensile strength and (**b**) elongation at break.

A prerequisite for an ANOVA is a normal distribution of the dependent variable. For this purpose, 20 samples with the same process settings serve as a random sample to determine the statistical distribution of tensile strength and elongation at break. The normal Q-Q plot can be used for a graphical interpretation if the results of the tensile tests follow a normal distribution. If this is the case, the observed values are approximately at the theoretically expected values and therefore on a diagonal. Figure 9 shows the normal Q-Q plot for tensile strength and elongation at break. The results of both target values seem to fit the standard normal quite well, but slightly tailed for the tensile strength and higher values of elongation at break around the mean value. Due to the rather subjective interpretation of a Q-Q plot, especially with a small number of samples, the qualitative method of the Anderson–Darling test is also used to test for normal distribution. A *p*-value < 0.05 corresponds to a significant deviation from a normal distribution. With a *p*-value of 0.36, it can therefore not be ruled out that the tensile strength follows a normal distribution. In contrast, the elongation at break appears to show a significant deviation from a normal distribution with a *p*-value of 0.03, despite the positive first impression of the Q-Q plot. Nevertheless, subsequent ANOVA is performed for the tensile strength as well as for the elongation at break. Several studies [36,37,38,39,40] emphasize the robustness of an ANOVA in the case of a non-normally distributed sample. However, the following results for elongation at break should be viewed with particular caution.

### 3.4. ANOVA and Interactions

An ANOVA is performed to assess the significance of various effects on the respective objectives of tensile strength (Table 2), elongation at break (Table 3), and FVF (Table 4).

**Figure 10:**
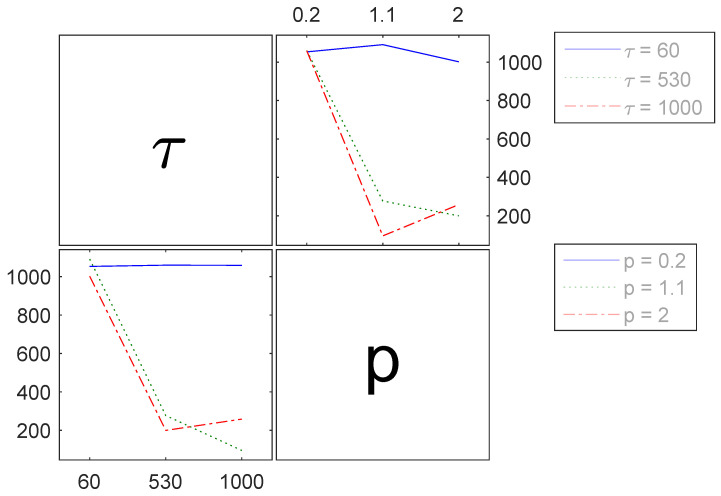
Interactions of the process parameters holding time τ in *s* and pressure *p* in MPa (abscissa of each graph) for the tensile strength σ in MPa (ordinate of each graph).

The interaction between holding time τ and pressure *p* with regard to tensile strength shows a very small *p*-value of $1.52\;\cdot \;10^{-16}$, which indicates a high level of significance. This is supported by much higher F-values for both the main effects and the interaction, compared to the critical F-value of 4.85 and 3.46, respectively. However, the significant main effects in Table 2 are questionable, particularly when considering the raw results depicted in Figure 7. In those results, alterations in tensile strength are not substantially apparent when only one parameter changes, contrary to the presence of a significant interaction. Conversely, when the main effects are not factored in, the F-value for the interaction decreases to 3.1, accompanied by a *p*-value to 0.02. Additionally, the accuracy of the regression model diminishes. Consequently, the main effects are retained within the ensuing regession model. The affected results concerning the interaction between holding time τ and pressure *p* are shown in Figure 10. Concurrent variations in both parameters causes a decrease in tensile strength (illustrated by the dotted and dash-dotted lines in Figure 10). Conversely, modifying just one parameter while maintaining the other at its lowest setting yields hardly any discernible effect on the target value (solid line in Figure 10).

The calculated *p*-value of 0.02, signifying the interaction among the observed process parameters for elongation at break (Table 3), also highlights its significant influence. As well as for the tensile strength, the presence of interaction between holding time and pressure, along with the similar trends in elongation at break when only one parameter is altered (Figure 8), suggests that debating the main effects might be unnecessary. Moreover, if the interaction is exclusively deemed relevant, the F-value decreases to 1.67 and the *p*-value decreases to 0.17. This adjustment would also lead to a regression model of lesser accuracy. As a result, the main effects continue to be considered for the subsequent regression model.

**Figure 11:**
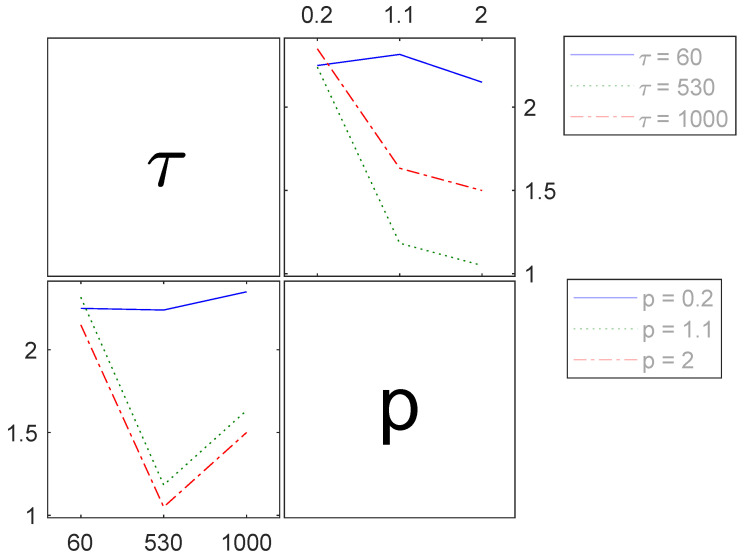
Interactions of the process parameters holding time τ in *s* and pressure *p* in MPa (abscissa of each graph) for the elongation at break $\epsilon_{b}$ in % (ordinate of each graph).

Figure 11 shows the interaction between the process parameters affecting the elongation at break. When one parameter is maintained at its lowest setting, there is hardly any noticeable effect on the target value. However, when the pressure is above 1.1 MPa and holding time increases from 530 s to 1000 s, a moderate increase in elongation at break is observed. Furthermore, an extended holding time of 1000 s seems to result in a relatively smaller reduction in the target value compared to the moderate setting of (530 s).

Nonetheless, for achieving high values of both tensile strength and elongation at break, opting for lower settings of both parameters is deemed preferable. This is in accordance with the macroscopic (Figure 3) and microscopic (Figure 4) view of the laminates. A simultaneous increase in the process pressure and the holding time leads to the fibres and matrix material being pressed out, which causes a displacement of the fibres to the 0°-axis and thus leads to premature failure of the composite. This finding is consisting with Christmann et al. [18] and Kropka et al. [25], but is contrary with other observations [10,26].

Regarding the FVF, no main effects or interaction of the process parameters are detected (Table 4). As detailed earlier, the impregnation quality and compaction of all laminates are excellent (Figure 4). This outcome is likely a consequence of the effective pre-impregnation of the UD-tapes, which in turn contributes to the minimal presence of microscopic voids prior to thermoforming. Additionally, the absence of substantial pressure requirement to eliminate macroscopic voids further attests to the quality of the prepreg material. Consequently, the quality of the prepreg may effect the objectives significantly, as proposed by Kropka et al. [25].

### 3.5. Regression Model and Validation

To predict the resulting mechanical properties of manufactured laminates, regression models are formulated based on the experimental and statistical evaluation of the process parameters. The highest possible values for tensile strength and elongation at break are aimed for within feasible process parameters. Equation (1) offers a solution for the tensile strength σ in MPa, depending on the holding time τ in *s* and the pressure *p* in MPa:

(1) σ=1088.4−0.1277·τ−79.4·p−0.443·τ·p

Equation (2) describes the predicted target value for the elongation at break $\epsilon_{b}$ in % affected by the process parameters:

(2) $$\epsilon_{b}=2.26+5\;\cdot \;10^{-5}\;\cdot \;\tau -0.16\;\cdot \;p-4\;\cdot \;10^{-4}\;\cdot \;\tau \;\cdot \;p$$

The subsequent process parameters are set to validate the model for predicting high values of tensile strength and elongation at break:

- holding time τ = 200 s
- pressure *p* = 0.3 MPa

A laminate is fabricated with the aforementioned parameters, followed by the execution of tensile tests. Table 5 presents a comparison between the measurements acquired from the validation samples and the corresponding predicted results. The calculated values exhibit a strong correspondence with the measured results. Both the tensile strength and elongation of the validation samples fall within the confidence intervals (CI) established by the model, while the means are slightly above the predictions. The percentage error between the predicted and measured mean values remains notably low, at 4.8% for the tensile strength and 8.7% for the elongation at break.

## 4. Conclusions

This paper demonstrates the efficacy of employing statistical methods as a suitable approach for assessing the effects of process parameters in the context of UD-tape thermoforming. A strong interaction is observed between holding time and pressure, resulting in a sudden decrease in tensile strength and elongation at break due to fibre misalignment, which occurs when both parameters are concurrently elevated. This confirms the results of previous studies, which were determined using other methods [18,25]. However, the fibre volume fraction remains unaffected by the process parameters, possibly due to the effective pre-impregnation of the UD-tapes. Further research endeavours will incorporate additional input variables, such as prepreg quality, process temperature, varying matrix materials, as well as multi-level variations of holding time and pressure. This approach would foster a more comprehensive understanding of the effects of relevant parameters on the thermoforming process.

## Figures and Tables

**Table 1:** Parameters of the thermoforming process for a full factorial desgin matrix.

Laminate	Holding Time τ in s	Pressure *p* in MPa
A	60	0.2
B	60	1.1
C	60	2.0
D	530	0.2
E	530	1.1
F	530	2.0
G	1000	0.2
H	1000	1.1
I	1000	2.0

**Table 2:** ANOVA of the tensile strength σ.

Effect	Sum of Squares	Degrees of Freedom	Mean Square	F-Value	*p*-Value
Holding time	$3.72\;\cdot \;10^{6}$	2	$1.86\;\cdot \;10^{6}$	194.42	$7.20\;\cdot \;10^{-23}$
Pressure	$3.94\;\cdot \;10^{6}$	2	$1.97\;\cdot \;10^{6}$	206.07	$2.22\;\cdot \;10^{-23}$
Interaction	$2.05\;\cdot \;10^{6}$	4	$5.12\;\cdot \;10^{5}$	53.56	$1.52\;\cdot \;10^{-16}$
Error	$4.30\;\cdot \;10^{5}$	45	$9.57\;\cdot \;10^{3}$	–	–
Total	$1.01\;\cdot \;10^{7}$	53	–	–	–

**Table 3:** ANOVA of the elongation at break $\epsilon_{b}$.

Effect	Sum of Squares	Degrees of Freedom	Mean Square	F-Value	*p*-Value
Holding time	4.97	2	2.49	11.46	$9.52\;\cdot \;10^{-5}$
Pressure	5.41	2	2.71	12.47	$4.91\;\cdot \;10^{-5}$
Interaction	2.75	4	0.69	3.16	0.02$\;\cdot \;10^{-5}$
Error	9.77	45	0.22	–	–
Total	22.89	53	–	–	–

**Table 4:** ANOVA of the fibre volume fraction (FVF).

Effect	Sum of Squares	Degrees of Freedom	Mean Square	F-Value	*p*-Value
Holding time	21.93	2	10.96	0.89	0.42
Pressure	61.59	2	30.80	2.50	0.09
Interaction	34.19	4	8.55	0.69	0.60
Error	554.17	45	12.31	–	–
Total	671.87	53	–	–	–

**Table 5:** Results of the validation for the tensile strength σ and elongation at break $\epsilon_{b}$ with the mean values and standard deviation (SD) compared to the model’s prediction and the corresponding confidence interval (CI) as well as the error in % of the means and predictions.

	Mean	SD	Prediction	CI	Error in %
σ in MPa	1049	±40	1012	±201	3.7
$\epsilon_{b}$ in %	2.3	±0.6	2.2	±0.4	4.5

## Data Availability

Data are contained within the article.

## Abbreviations

The following abbreviations are used in this manuscript:

ANOVA	Analysis of variance
BF	Basalt fibre
CI	Confidence interval
DOE	Design of experiments
FRP	Fibre reinforced plastic
FRTP	Fibre reinforced thermoplastic
FVF	Fibre volume fraction
PA 6	Polyamide 6
PEEK	Polyether ether ketone
Prepreg	Pre-impregnated material
SEM	Selective electron microscopy
SD	Standard deviation
UD-tape	Unidirectional fibre-reinforced tape
